# Prevalence and health care costs of mitochondrial disease in Ontario, Canada: A population-based cohort study

**DOI:** 10.1371/journal.pone.0265744

**Published:** 2022-04-08

**Authors:** Emmalin Buajitti, Laura C. Rosella, Ersi Zabzuni, L. Trevor Young, Ana C. Andreazza

**Affiliations:** 1 Dalla Lana School of Public Health, University of Toronto, Toronto, Ontario, Canada; 2 ICES, Toronto, Ontario, Canada; 3 Institute for Better Health, Trillium Health Partners, Mississauga, Ontario, Canada; 4 Department of Laboratory Medicine and Pathology, Faculty of Medicine, University of Toronto, Toronto, Ontario, Canada; 5 Department of Pharmacology and Toxicology, University of Toronto, Toronto, Ontario, Canada; 6 Department of Psychiatry, Faculty of Medicine, University of Toronto, Toronto, Ontario, Canada; 7 Mitochondrial Innovation Initiative, MITO2i, University of Toronto, Toronto, Ontario, Canada; Chang Gung Memorial Hospital and Chang Gung University, Taoyuan, Taiwan, TAIWAN

## Abstract

**Background:**

Mitochondrial disease prevalence has been estimated at 1 in 4000 in the United States, and 1 in 5000 worldwide. Prevalence in Canada has not been established, though multi-linked health administrative data resources present a unique opportunity to establish robust population-based estimates in a single-payer health system. This study used administrative data for the Ontario, Canada population between April 1988 and March 2019 to measure mitochondrial disease prevalence and describe patient characteristics and health care costs.

**Results:**

3069 unique individuals were hospitalized with mitochondrial disease in Ontario and eligible for the study cohort, representing a period prevalence of 2.51 per 10,000 or 1 in 3989. First hospitalization was most common between ages 0–9 or 50–69. The mitochondrial disease population experiences a high need for health care and incurred high costs (mean = CAD$24,023 in 12 months before first hospitalization) within the single-payer Ontario health care system.

**Conclusions:**

This study provides needed insight into mitochondrial disease in Canada, and demonstrates the high health burden on patients. The methodology used can be adapted across jurisdictions with similar routine collection of health data, such as in other Canadian provinces. Future work should seek to validate this approach via record linkage of existing disease cohorts in Ontario, and identify specific comorbidities with mitochondrial disease that may contribute to high health resource utilization.

## Introduction

Dysfunction of the mitochondria caused by mutations in nuclear DNA or mitochondrial DNA can result in a group of disorders known as mitochondrial disease [1]. It can present itself during childhood or adulthood and over 250 genes have been found to be implicated in this disease [2]. Although genetic targets have been identified, mitochondrial disease presents inter-individual heterogeneity and can be challenging to diagnose [2]. Mitochondrial disease has also been shown to present with comorbidities, such as diabetes and Parkinson’s disease [3]. This is due to the high abundance of mitochondria in virtually every cell of the body. Furthermore, evidence suggests that mitochondrial disease presents a large economic burden on the healthcare system and patients. In the United States, the average cost per month for mitochondrial disease treatment has been estimated between $3100 and $4829, considerably higher than the average healthcare costs in the population [4].

The first diagnosis of mitochondrial disease was in 1959, making it a fairly novel discovery [5]. Its prevalence has been studied using cohorts based on clinical and/or administrative health data in Europe, Australia and North America [4, 6–9]. A study from the North East of England determined prevalence in this region by ascertaining all adults with symptoms of mitochondrial disease were referred to the same clinic (i.e., The Newcastle Mitochondrial Centre) [7]. In the United States, mitochondrial disease prevalence has been reported as 1 in 4000 [10], and 1 in 5000 globally [11]. However, the prevalence in Canada remains unexplored, in part because of province- and territory-specific health care systems and variation in billing practices and codes across provinces. Generating robust prevalence estimates in Ontario, Canada’s most populous province, will make important contributions to our understanding of patients experiencing mitochondrial disease and provide evidence about the needs of this complex patient population in Ontario’s single-payer health care system. These methods can be translated across provinces and to other health settings with similar routine collection of health data.

The objective of this study was to determine the prevalence of mitochondrial disease in Ontario, Canada. We leveraged comprehensive, multi-linked health administrative data in a single-payer health system from April 1988 to March 2019 to identify all patients hospitalized with mitochondrial disease diagnoses in Ontario over a 30-year period. We used demographic and health care data to construct prevalence estimates and describe patient characteristics in the mitochondrial disease population.

## Materials and methods

### Data sources

This study used multiple databases linked at ICES, a research institute that holds health administrative data records for individuals eligible for Ontario’s single-payer health insurance system (OHIP) at any point since 1988. Generally speaking, ICES data are available for nearly all Ontario residents for any period in which they receive care through OHIP. The centralized registry of OHIP-eligible individuals is known as the Registered Persons’ Database or RPDB. RPDB also contains basic client demographics, including sex, age, and postal code of residence.

Cases of mitochondrial disease were identified using hospitalization records from the Discharge Abstract Database (DAD), which captures acute care hospitalizations (inpatient stays) from April 1988 to March 2019.

Health care costs were based on complete ICES administrative data holdings, encompassing health care encounters including hospitalizations (DAD), emergency department visits (National Ambulatory Care Reporting System), physician billings, drug payments (Ontario Drug Benefit), home care and long-term care, and others. A full list of databases used is available in the published methodological guidelines [12].

### Cohort identification

Individuals with mitochondrial disease defined as those with one or more inpatient hospitalizations with a diagnostic code indicating mitochondrial disease. Between April 1988 and March 2002, the International Classification of Diseases (ICD)9 code 277.8 was used. In 2002, Ontario hospital billings transitioned to ICD-10-CA, a Canadian modified version of ICD-10 [13]. From April 2002 to March 2019, ICD-10 code G71.3 was used to identify diagnoses of mitochondrial disease.

Individuals with one or more mitochondrial disease-related admissions were excluded if they could not be linked to a valid record in RPDB, or were not OHIP eligible for at least 12 months pre- and post-admission. The reason for the eligibility exclusion is to ensure that individuals were receiving most of their health care in Ontario before and after hospitalization.

Period prevalence of mitochondrial disease (1988 to 2019) per 1000 was calculated by dividing the total number of identified patients by the Ontario population in the midpoint year (2003).

Cohort entries were grouped according to the calendar year of first hospital admission. Sex and age were identified based on individuals’ RPDB record at time of first hospital admission.

### Health care costs

Health care costs incurred for a 12-month period before and after hospitalization were estimated using a costing methodology developed at ICES, which has been defined in detail elsewhere [12]. Briefly, the costs are based on identifying every health care interaction for a given individual captured by ICES data holdings. Each interaction is then assigned a cost based on billing data and known fiscal expenditures. Finally, costs are aggregated across the 12-month period to produce a summary of health care expenditures for each member of the study cohort. Costs of the index hospitalization were excluded from both time periods. Because costing data are only available for fiscal years 2002 and later, health care costs were computed only for those whose index admission took place on April 1, 2003 or after.

### Ethics approval

This study was approved by the University of Toronto Health Sciences Research Ethics Board.

## Results

### Prevalence of mitochondrial disease

**Fig 1:**
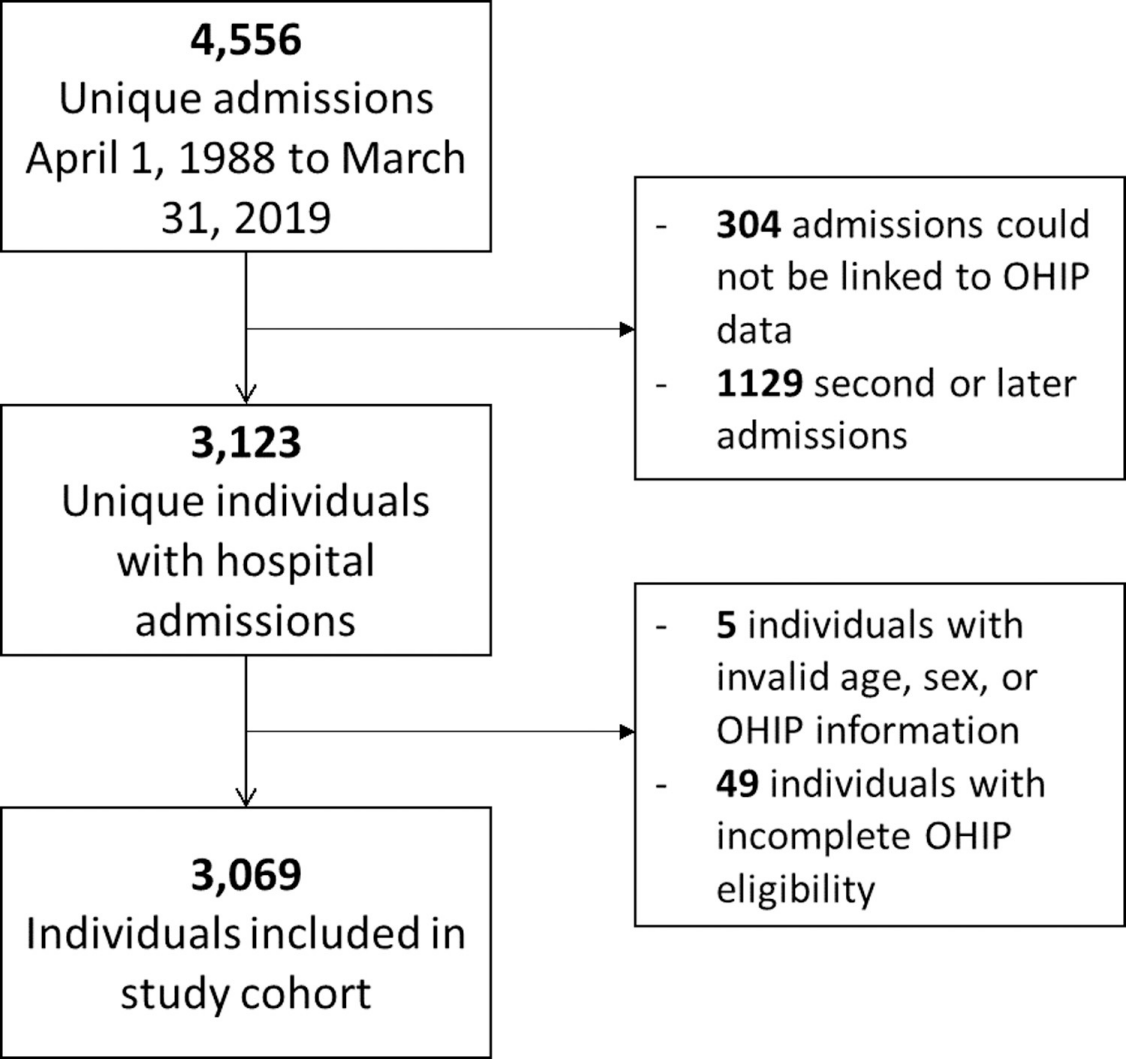
Study inclusion flow chart.

A flow chart depicting cohort inclusions and exclusions is shown in Fig 1. Overall, 3123 unique individuals had one or more hospitalizations with a diagnostic code indicating mitochondrial disease and could be linked to a valid Registered Persons’ Database (RPDB) record. After exclusions for incomplete Ontario Health Insurance Plan (OHIP) eligibility or missing demographic information, 3069 individuals were included in the study cohort. Denominators were based on a midpoint year population equal to 12,243,758. The overall period prevalence of mitochondrial disease was identified as 2.51 cases per 10,000, or 1 in 3989. This represents a conservative lower bound of mitochondrial disease prevalence in Ontario since diagnoses that took place in outpatient settings are not captured.

**Fig 2:**
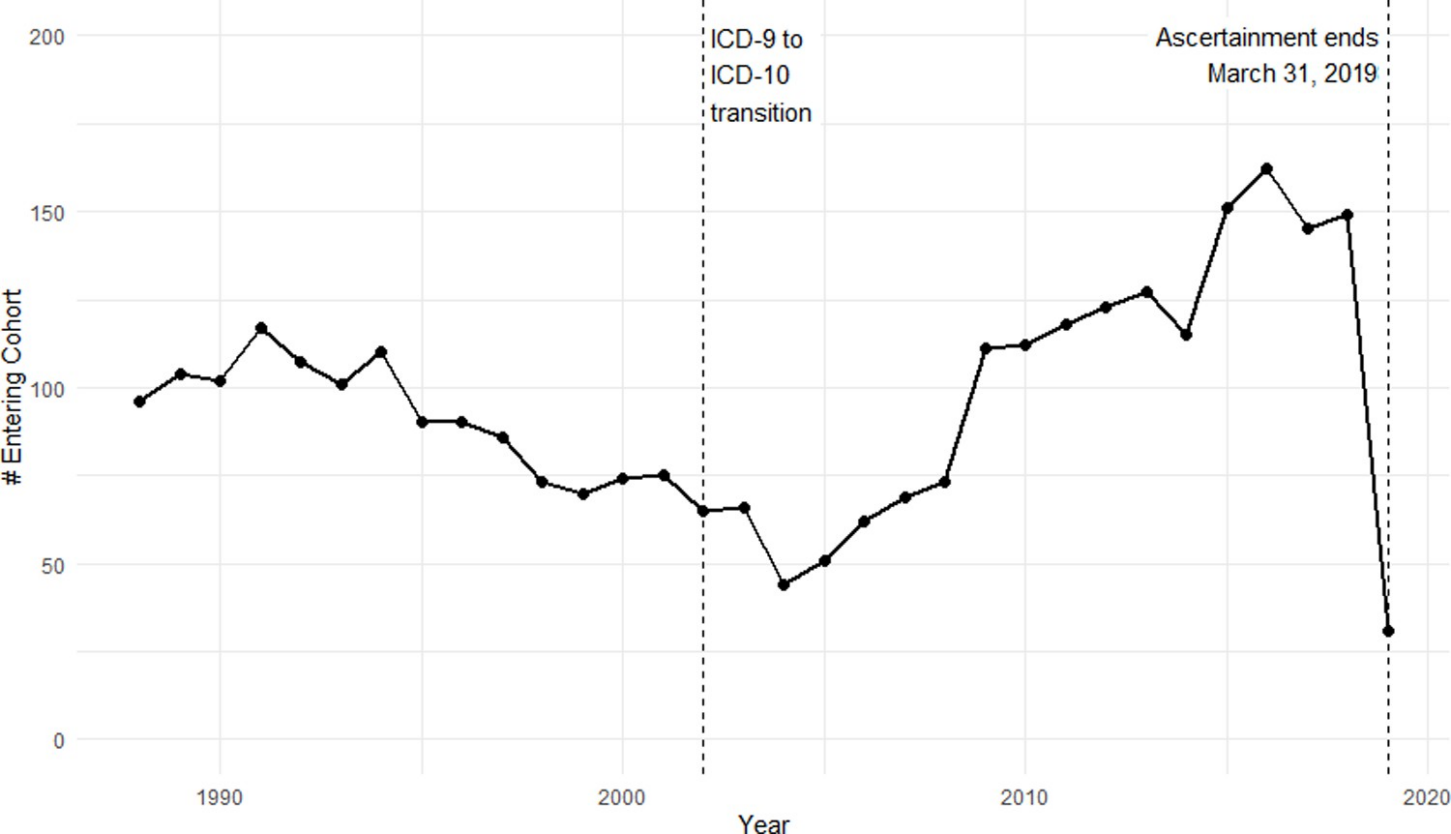
Frequency of mitochondrial disease by year of entry into study cohort, 1988 to 2019. 2019 is incomplete because of study termination on March 31, 2019.

Fig 2 shows the number of new cohort entries by calendar year. There is a V-shaped trend in study cohort entries, with declining counts between 1988 and 2004, and increasing counts from 2004 to 2019 (note that 2019 is incomplete because of study termination on March 31, 2019). The date of cohort entry (defined as the first hospital admission date) may or may not represent the date at which a mitochondrial disease patient receives or becomes aware of their diagnosis.

### Cohort characteristics

Table 1 shows the demographic characteristics of the study cohort. There were no major sex differences in the study population. In terms of age at cohort entry, first hospitalization was most common between ages 0–9, with 19 percent of the study population entering the cohort before age 10. A substantial number of cohort entries also took place in older ages, with 31.5 percent entering the cohort between ages 50 and 69.

**Table 1:** Study cohort characteristics (n = 3069).

Variable	Category	n	%
Sex	M	1435	46.8
	F	1634	53.2
Age at cohort entry	0–9	583	19.0
	10–19	224	7.3
	20–29	195	6.4
	30–39	240	7.8
	40–49	363	11.8
	50–59	452	14.7
	60–69	515	16.8
	70–79	343	11.2
	80+	154	5.0

### Health care costs

Health care costs incurred by members of the study population before and after their index hospitalization are shown in Table 2. This analysis was limited to those with hospital admissions on or after April 1, 2002 (n = 1695; see Methods).

**Table 2:** Health care costs incurred 12 months before and after hospitalization (n = 1695).

Summary measure	mean	SD	median	minimum	maximum
Cost before hospitalization (CAD)	24023	40075	9839	727	414661
Cost after hospitalization (CAD)	33545	52829	11445	406	565424

Overall, health care costs in this population were high both before and after mitochondrial disease-related hospitalization. The mean cost incurred in the 12 months prior to hospitalization was $24,023 (median = $9,839) and the mean cost incurred in 12 months post-discharge was $33,545 (median = $11,445).

## Discussion

### Key findings

Overall, we identified 3069 unique individuals hospitalized with mitochondrial disease in Ontario, Canada, between 1988 and 2019 and found a period prevalence of 2.5 cases per 10,000 between 1988 and 2019 (1 in 3989). This prevalence estimate is very similar to cited prevalence estimates of 1 in 4000 in the United States [10], and somewhat higher than the 1 in 5000 prevalence estimated globally [11]. Since cases were restricted to those experiencing hospitalization, this estimate is likely a conservative lower bound on mitochondrial disease prevalence in the Ontario population.

We used the midpoint year (2003) population of 12,243,758 to calculate our prevalence estimate. We used the midpoint population as it is a routinely used denominator for period prevalence calculations and approximately reflects the average population across the study period [14]. However, the Ontario population is dynamic and ranged from 9,838,620 to 14,544,701 in our study period (1988 to 2018). Our prevalence estimate would therefore have varied based on the population denominator used–for instance, from 1 in 3206 to 1 in 4739 based on those population extremes.

Our study found decreasing mitochondrial disease cases between 1988 and 2004, and increasing cases after 2004. The trend reversal coincides somewhat with the transition from International Classifications of Diseases (ICD) 9 to ICD-10-CA, which took place in March 2002. Thus it is possible that diagnostic coding has influenced observed trends in mitochondrial disease-related hospitalizations. Sensitivity analyses were carried out to determine whether case composition changed as a result of the ICD-9 to ICD-10-CA transition; that is, whether individuals captured by ICD-9 coding were systematically different from those captured by ICD-10-CA coding. The results of this sensitivity analysis, which are shown in full in S1 File, suggest that there is no demographic difference between the groups, and it is unlikely that coding changes have substantially biased observed diagnostic trends.

Our study findings also demonstrate that the mitochondrial disease population experiences a high need for health care and incur high costs within the health care system. Health care costs were higher in the 12 months following mitochondrial disease-related hospitalization, compared to the 12 months before hospitalization. Because the cost of the index hospitalization was excluded from these calculations, this increase cannot be attributed to the cost of hospitalization itself, although it may be related to post-hospitalization care (e.g. rehabilitation or specialist follow-up expenses).

For comparison, a study of high-cost health system users in Ontario between 2009 and 2011 found a median health care cost of $333, with only 5% of health system users incurring $7,961 or more of health care costs per year [15]. Based on this cutoff, over half of our study cohort would have been classified as high-cost users in Ontario’s single-payer system in the 12 months prior to their index hospitalization. This is consistent with findings from an insured population in the United States, where mitochondrial disease patients incurred significantly more health care claims compared to the general population [4]. Other research in the US also found high burden of morbidity, health care costs, and excess mortality in a mitochondrial disease population similar to other complex patients (including those with Friedreich’s Ataxia and muscular dystrophy) [16]. It is significant to note that our findings were similar despite the substantial differences in health care systems in Ontario and the United States.

### Limitations

This prevalence study is limited to hospitalized cases of mitochondrial disease as a result of diagnostic code limitations in the Ontario health administrative data. Ontario outpatient physician fee billings are based on a modified ICD-9 coding system and are not coded with sufficient granularity to identify rare diseases such as mitochondrial disease. Patients who have received only outpatient care (e.g. specialist clinic) may be aware of their mitochondrial disease diagnosis, but could not be captured by our data. As a result, it is likely that our study population captures only more severe cases of mitochondrial disease or individuals with severe comorbid conditions (who would be hospitalized at some point in their care). Our prevalence estimates should thus be interpreted in the context of this low-sensitivity, high-specificity approach; it is likely that true prevalence of mitochondrial disease is greater than observed as a result of missing cases who were never hospitalized.

Another limitation of this approach is that the date of cohort entry is based on the first hospitalization instance, and may or may not coincide with the patient receiving their mitochondrial disease diagnosis. We are thus unable to make direct conclusions regarding cohort age at first diagnosis. Specifically, patients in older age groups at time of cohort entry may have received their diagnosis in younger ages, but could not be identified in our data until hospitalization was necessitated either by escalating symptoms or acute care needs related to comorbid chronic disease (which tend to escalate with age). While we believe that age at cohort entry provides some relevant insight into the age distribution of the mitochondrial disease population, these findings should be interpreted with caution in light of this limitation.

Because our study used health administrative data, we were constrained to available variables. This limited our analysis of the data as we lacked information about clinical and family history; unfortunately, there is no comprehensive data resource in Ontario or Canada which captures clinical presentations for the entire population. We feel strongly that our analysis adds value for the understanding of mitochondrial disease in the Canadian population, but acknowledge the constrained scope of our research and findings in this study.

Finally, we are unable to directly verify coding practices for mitochondrial disease among all Ontario physicians. The diagnostic codes we used were based on previous, validated studies–however, because Canada uses a specialized modification of the ICD-10 system (ICD-10-CA), it is possible that coding practices are different from those international jurisdictions. We did confirm our approach with specialists working directly with mitochondrial disease patients and feel confident in its appropriateness.

## Conclusions

Our study is an early step in understanding the prevalence of mitochondrial disease in Ontario, Canada, along with the health burden on patients as well as Ontario’s single-payer health care system. By leveraging comprehensive population-based health care records, this preliminary study provides much-needed empirical evidence about the prevalence of mitochondrial disease in Ontario between 1988 and 2019. In addition, our mitochondrial disease cohort clearly demonstrates the high level of health care need in this population; future work should seek to identify specific comorbidities with mitochondrial disease that may contribute to high health resource utilization.

Our proposed methodology can be adapted across Canada; specifically, British Columbia and Manitoba have similar linked data resources [17, 18]. Other surveillance systems in Canada, such as the National Diabetes Surveillance System [19], have successfully leveraged administrative data from across provinces–a similar approach may be viable for monitoring mitochondrial disease inter-provincially. Additionally, formal validation via record linkage of existing disease cohorts in Ontario could validate our approach in the Ontario context [20].

Working towards a validated disease cohort will provide significant future opportunity for research on the epidemiology and health service utilization of mitochondrial disease. Understanding of the prevalence and patient population of mitochondrial disease adds important value to our knowledge of the epidemiology of this disease. Further understanding of the burden of this disease and its co-morbidities on both patients and the health care system can support better planning to close gaps in care and improve delivery of health services to mitochondrial disease patients.

## Supporting information

S1 File Comparison of cases ascertained using ICD-9 and ICD-10 codes. (DOCX)

## References

[1] Ng YS, Turnbull DM. Mitochondrial disease: genetics and management. J Neurol. 2016;263(1):179–91. doi: 10.1007/s00415-015-7884-3 26315846 PMC4723631

[2] Alston CL, Rocha MC, Lax NZ, Turnbull DM, Taylor RW. The genetics and pathology of mitochondrial disease. J Pathol. 2017;241(2):236–50. doi: 10.1002/path.4809 27659608 PMC5215404

[3] Catalán-García M, García-García FJ, Moreno-Lozano PJ, Alcarraz-Vizán G, Tort-Merino A, Milisenda JC, et al. Mitochondrial Dysfunction: A Common Hallmark Underlying Comorbidity between sIBM and Other Degenerative and Age-Related Diseases. Journal of Clinical Medicine. 2020;9(5):1446. doi: 10.3390/jcm9051446 32413985 PMC7290779

[4] Cohen B, Balcells C, Hotchkiss B, Aggarwal K, Karaa A. A retrospective analysis of health care utilization for patients with mitochondrial disease in the United States: 2008–2015. Orphanet Journal of Rare Diseases. 2018;13(1):210. doi: 10.1186/s13023-018-0949-5 30466460 PMC6251171

[5] Koenig MK. Presentation and diagnosis of mitochondrial disorders in children. Pediatric neurology. 2008;38(5):305–13. doi: 10.1016/j.pediatrneurol.2007.12.001 18410845 PMC3099432

[6] Arpa J, Cruz‐Martínez A, Campos Y, Gutiérrez‐Molina M, García‐Rio F, Pérez‐Conde C, et al. Prevalence and progression of mitochondrial diseases: a study of 50 patients. Muscle & nerve. 2003;28(6):690–5. doi: 10.1002/mus.10507 14639582

[7] Gorman GS, Schaefer AM, Ng Y, Gomez N, Blakely EL, Alston CL, et al. Prevalence of nuclear and mitochondrial DNA mutations related to adult mitochondrial disease. Annals of neurology. 2015;77(5):753–9. doi: 10.1002/ana.24362 25652200 PMC4737121

[8] Manwaring N, Jones MM, Wang JJ, Rochtchina E, Howard C, Mitchell P, et al. Population prevalence of the MELAS A3243G mutation. Mitochondrion. 2007;7(3):230–3. doi: 10.1016/j.mito.2006.12.004 17300999

[9] Schaefer AM, McFarland R, Blakely EL, He L, Whittaker RG, Taylor RW, et al. Prevalence of mitochondrial DNA disease in adults. Annals of Neurology: Official Journal of the American Neurological Association and the Child Neurology Society. 2008;63(1):35–9. doi: 10.1002/ana.21217 17886296

[10] Ahuja AS. Understanding mitochondrial myopathies: a review. PeerJ. 2018;6:e4790. doi: 10.7717/peerj.4790 29844960 PMC5967365

[11] Thorburn DR. Mitochondrial disorders: Prevalence, myths and advances. Journal of Inherited Metabolic Disease. 2004;27(3):349–62. doi: 10.1023/B:BOLI.0000031098.41409.55 15190193

[12] Wodchis WP, Bushmeneva K, Nikitovic M, McKillop I. Guidelines on person-level costing using administrative databases in Ontario. 2013.

[13] Walker RL, Hennessy DA, Johansen H, Sambell C, Lix L, Quan H. Implementation of ICD-10 in Canada: how has it impacted coded hospital discharge data? BMC health services research. 2012;12(1):1–9. doi: 10.1186/1472-6963-12-149 22682405 PMC3411494

[14] Spronk I, Korevaar JC, Poos R, Davids R, Hilderink H, Schellevis FG, et al. Calculating incidence rates and prevalence proportions: not as simple as it seems. BMC Public Health. 2019;19(1):512. doi: 10.1186/s12889-019-6820-3 31060532 PMC6501456

[15] Wodchis WP, Austin PC, Henry DA. A 3-year study of high-cost users of health care. Cmaj. 2016;188(3):182–8. doi: 10.1503/cmaj.150064 26755672 PMC4754179

[16] McCormack SE, Xiao R, Kilbaugh TJ, Karlsson M, Ganetzky RD, Cunningham ZZ, et al. Hospitalizations for mitochondrial disease across the lifespan in the U.S. Mol Genet Metab. 2017;121(2):119–26. doi: 10.1016/j.ymgme.2017.04.007 28442181 PMC5492979

[17] Dart AB, Martens PJ, Sellers EA, Brownell MD, Rigatto C, Dean HJ. Validation of a pediatric diabetes case definition using administrative health data in Manitoba, Canada. Diabetes care. 2011;34(4):898–903. doi: 10.2337/dc10-1572 21378211 PMC3064048

[18] Sadatsafavi M, Lynd L, Marra C, Carleton B, Tan WC, Sullivan S, et al. Direct health care costs associated with asthma in British Columbia. Canadian respiratory journal. 2010;17(2):74–80. doi: 10.1155/2010/361071 20422063 PMC2866215

[19] LeBlanc AG, Jun Gao Y, McRae L, Pelletier C. At-a-glance—Twenty years of diabetes surveillance using the Canadian Chronic Disease Surveillance System. Health Promot Chronic Dis Prev Can. 2019;39(11):306–9. doi: 10.24095/hpcdp.39.11.03 31729313 PMC6876649

[20] Heighton JN, Brady LI, Newman MC, Tarnopolsky MA. Clinical and demographic features of chronic progressive external ophthalmoplegia in a large adult-onset cohort. Mitochondrion. 2019;44:15–9. doi: 10.1016/j.mito.2017.12.006 29246868

