# Supplementary material for: Prevalence and health care costs of mitochondrial disease in Ontario, Canada: A population-based cohort study
Source: PLoS One. 2022 Apr 8;17(4):e0265744. doi: 10.1371/journal.pone.0265744 (PMC8993002; doi:10.1371/journal.pone.0265744)
Supplement: S1 File — (DOCX) [file pone.0265744.s001.docx]

**S1 Supporting Information. Comparison of cases ascertained using ICD-9 and ICD-10 codes.**

**Table A.** Characteristics of mitochondrial disease cases ascertained using ICD-9 codes, April 1988 to March 2002 (n=1313).

| **Variable** | **Category** | **n** | **%** |
| --- | --- | --- | --- |
| Sex | M | 619 | 47.1 |
|  | F | 694 | 52.9 |
| Age at cohort entry | 0-9 | 260 | 19.0 |
|  | 10-19 | 96 | 7.3 |
|  | 20-29 | 101 | 7.7 |
|  | 30-39 | 122 | 9.3 |
|  | 40-49 | 168 | 12.8 |
|  | 50-59 | 163 | 12.4 |
|  | 60-69 | 218 | 16.6 |
|  | 70-79 | 148 | 11.2 |
|  | 80+ | 37 | 2.8 |

**Table B.** Characteristics of mitochondrial disease cases ascertained using ICD-10 codes, April 2002 to March 2019 (n=1756).

| **Variable** | **Category** | **n** | **%** |
| --- | --- | --- | --- |
| Sex | M | 816 | 46.5 |
|  | F | 940 | 53.5 |
| Age at cohort entry | 0-9 | 323 | 18.4 |
|  | 10-19 | 128 | 7.3 |
|  | 20-29 | 94 | 5.4 |
|  | 30-39 | 118 | 6.7 |
|  | 40-49 | 195 | 11.1 |
|  | 50-59 | 289 | 16.5 |
|  | 60-69 | 297 | 16.9 |
|  | 70-79 | 195 | 11.1 |
|  | 80+ | 117 | 6.7 |
